# The ectomycorrhizal community of urban linden trees in Gdańsk, Poland

**DOI:** 10.1371/journal.pone.0237551

**Published:** 2021-04-26

**Authors:** Jacek Olchowik, Marzena Suchocka, Paweł Jankowski, Tadeusz Malewski, Dorota Hilszczańska

**Affiliations:** 1 Department of Plant Protection, Institute of Horticultural Sciences, Warsaw University of Life Sciences, Warsaw, Poland; 2 Department of Landscape Architecture, Institute of Environmental Engineering, Warsaw University of Life Sciences, Warsaw, Poland; 3 Department of Computer Information Systems, Institute of Information Technology, Warsaw University of Life Sciences, Warsaw, Poland; 4 Department of Molecular and Biometric Techniques, Museum and Institute of Zoology, Polish Academy of Science, Warsaw, Poland; 5 Department of Forest Ecology, Forest Research Institute, Sękocin Stary, Poland; Friedrich Schiller University, GERMANY

## Abstract

The linden tree (*Tilia* spp.) is a popular tree for landscaping and urban environments in central and northwest European countries, and it is one of the most popular in cities in Poland. Ectomycorrhizal fungi form a symbiosis with many urban tree species and protect the host plant from heavy metals and against salinity. The aim of this study was to characterise the ECM fungal community of urban linden trees along the tree damage gradient. The study was performed on two sites located in the centre of the city of Gdańsk, in northern Poland. The vitality assessment of urban linden trees was made according to Roloff’s classification. Tree damage classes were related to soil characteristics using principal component analysis. The five ectomycorrhizal fungal species were shared among all four tree damage classes, and *Cenococcum geophilum* was found to be the most abundant and frequent ectomycorrhizal fungal species in each class. Soil samples collected in the vicinity of trees belonging to the R0 class had significantly lower pH Na, Cl and Pb content than other soils. Our knowledge of ectomycorrhizal communities in urban areas is still limited, and these findings provide new insights into ectomycorrhizal distribution patterns in urban areas.

## Introduction

The most heavily human-modified ecosystems, cities, are expanding rapidly [1]. City managers are obliged to ensure sustainability and environmental benefits for city dwellers, so they introduce trees as a valuable component of an urban ecosystem [2]. Nevertheless, paradoxically, they grow in often extremely distorted habitat conditions in comparison to natural conditions. Street trees are exposed to a relatively high stress level. Studies reveal that their average lifespan is shorter than that of park trees [3], with mean ranging from 19 to 28 years [4] or less. Park trees are expected to grow in average 160 years.

The linden tree (*Tilia* spp.) is a popular tree for landscaping and urban environments in central and northwest European countries [5], and it is one of the most popular urban tree in cities in Poland. Dmuchowski and Badurek [6] reported that in Warsaw during 1973–2000 over 50% of trees growing alongside the four main thoroughfares in the city centre were removed. Moreover, the continuation of these studies has shown that over a period of 35 years, out of the 5 species with the highest loss, 3 were *Tilia* species: *Tilia platyphyllos*, *Tilia ‘Euchlora’* and *Tilia cordata* [7].

Stress conditions that affect urban trees may be of biotic or abiotic character, mechanical damage, high temperature, soil compaction, limited soil volume for root development and drought [3, 8, 9]. Specifically, soil and roots may be affected by construction activities such as utility trenching, soil compaction and subsequent root deoxygenation, shortage of available water, and incorporation of anthropic materials [5, 10, 11]. Under stress, plant growth and photosynthesis are reduced and carbon allocation is altered, resulting in a low tree vitality [12–17].

Mycorrhiza is a mutualistic association because fungi form relationships in and on the roots of a host plant. Mycorrhizae protect the host plant from heavy metals and against drought [18]. Ectomycorrhizal fungi (ECM) are ecologically significant because they provide the plant with several benefits, including enhanced nutrients [18] and increased water use efficiency, and enhanced root exploration. Mycorrhizal colonisation has been shown to promote short root survival, particularly when *Tilia* trees are exposed to drought conditions. Ectomycorrhizal fungi promote water uptake in general [e.g., 19] and have been specifically shown to play an important role in the nutrient uptake of *Tilia* spp. [20]. Mycorrhizae protect the host plant from heavy metals and promote short root survival, particularly when *Tilia* trees are exposed to drought conditions [18, 12]. It has been reported that this symbiosis plays a major role by increasing the efficiency of sodium-excluding mechanisms in infected roots and through higher root accumulation of phosphorus [21]. The fungi’s ecological distribution is markedly different from natural and urban environments, where mycorrhizal fungi have evolved and adapted. For example, Timonen and Kauppinen [22] reported that *Tilia cordata* trees growing in a nursery had different sets of ectomycorrhizal symbionts than trees grown along streets with traffic. However, the relationship between specific environmental conditions and the mycorrhizal status of trees is still not well known [23]. Taking into account the positive impact of the mycorrhizal relationship for trees performance we investigated the differences of ECM community of trees growing in the park (the favorable habitat conditions) and the trees growing along the streets.

The aim of this study was to characterise the ECM fungal community of urban linden trees along the tree damage gradient. We hypothesised that ECM fungal diversity would be the highest on least damaged trees.

## Materials and methods

### Study sites

We performed the study on two sites located in the centre of the city of Gdańsk, in northern Poland. The study was conducted on trees belonging to one genus, *Tilia*: Dutch Linden (*Tilia x europaea*), Fine Linden (*Tilia cordata*), and Broad-leaved Linden (*Tilia platyphyllos*). The first study area was located in the middle of Great Linden Avenue (54°22′05,5″N 18°37′51,2″E), which is a four-lane avenue created in 1768–1770 and located within the administrative borders of the City of Gdańsk. The avenue is located within one of the most important and busiest transport routes in Gdańsk. Great Linden Avenue is subject to legal protection under the Act of 16 April 2004 on Nature Conservation and the Act of 23 July 2003 on Monuments Protection and Care as an object entered in the register of monuments, no. 285 of 23.02.1967. By selecting a linden alley as the research area we excluded the variable of other trees, particularly deciduous species, affecting the community dynamics of *Tilia*-associated ECM fungi. The second site was located in a park (54°22′07,68″N 18°37′57,36″E) at a distance of approximately 150 m away and separated from the road by a dense strip of bushes and hedges.

### Tree health assessment

The vitality assessment of each tree was made according to Roloff’s classification [24] and the health condition of the trees was estimated according to leaf and branch growth pattern. The condition of each tree was evaluated based on distal crown vigour. Trees were segregated into 4 groups of different frequency: R0 ‘exploration’ (the phase of intensive offshoot growth), R1 ‘degeneration’ (a slightly delayed offshoot growth), R2 ‘stagnation’ (a visibly delayed offshoot growth), R3 ‘resignation’ (the tree is dying, no regeneration or return to the second class is possible). In the first study area, thirty street trees at least 200 m apart were classified according to the declining classes and assigned to classes R0, R1, R2 and R3. At the park site ten trees belonging to class RO were selected. Finally, 15, 6, 10 and 9 trees from the R0, R1, R2 and R3 Roloff classes were examined, respectively. All the trees situated along the street and in the park site were of the same age (60 years).

### Sampling and identification of mycorrhizae

In May 2019 soil cores were collected from both street and park trees (Field permit number: 18/A/2019/PZ, granted by City Hall of Gdańsk and Road and Greenery Department of Gdańsk). For each of 40 trees, a total of 80 soil subsamples were collected for mycorrhizal assessment: each sample consisted of 2 microsite localities: north and south (40 trees × 2 microsite (north and south) = 80 subsamples). The street root samples were taken from the 1.5 m wide grass strip between roadways. For the root system assessment, each sample was extricated with a cylinder (approximately 5 cm diameter, 20 cm depth) of the adjacent substrate and packed in labelled plastic bags. Samples were stored at– 20°C until further processing. All roots in each sample of equal volume were examined under a dissecting microscope at 10-60x magnification.

All root tips (100%) were classified as ‘vital ECM’ (VM, with ECM mantle) ‘non-vital’ (NV, scurfy surface, without remnants of ECM mantle) or ‘vital non-ECM’ (NM, well-developed, and mantle lacking) [25]. Mycorrhizae were classified into morphotypes based on morphological characters (colour, shape, texture, and thickness of the mantle, presence and organisation of the emanating hyphae, rhizomorphs, and other elements) according to Agerer [26], and the experience of the researchers involved in this study [27]. The degree of mycorrhization of linden roots, abundance, relative abundance and frequency of individual ectomycorrhizal fungal taxa were determined according to Olchowik et al. [27]. Each morphotype was treated separately during molecular identification and was pooled to calculate of abundance only after the molecular analysis indicated that morphotypes belonged to the same taxa. The internal transcribed spacer (ITS) region of the rDNA was amplified using the primers ITS1F and ITS4 [28, 29] and the product of the polymerase chain reaction (PCR) was sequenced. The full methods used for molecular identification of mycorrhizae are reported by Olchowik et al. [30]. The best representatives of each unique ITS sequences were deposited in NCBI GenBank with the following accession numbers: MT431581, MT431580, MT431583, MT431582, MT431579, MT431584, MT431587, MT431586, MT431585.

### Physicochemical analysis of the soil

The samples for soil chemical analysis were taken at the beginning of May 2019. The samples were collected from 30 trees in the alley and from the park area, which included 10 trees growing in the neighbouring area, within the boundaries of the city park. Samples of soil were air-dried, passed through a mesh screen, and stored for further analysis. The soil analyses were performed in the laboratory of the Polish Centre for Accreditation (No. AB312). The accuracy of the analysis was checked against standard reference materials: international standard soils [31–34]. The phosphorus (P) was determined for all samples with 1% citric acid extraction, according to Schlichting et al. [35]. The soil pH and was determined by mixing 20 ml of soil substrate with 40 ml of deionised water measured with a calibrated pH meter equipped with a glass electrode.

### Data analysis

For the purpose of data analysis, the two mycorrhizal data subsamples were summed for each tree to match the number of soil samples. Hence, a total of 40 samples were analysed in the study. All soil characteristics measured below the limit of detection were substituted with the half value of the corresponding limit. In order to investigate the relation between the tree damage classes and the abundance of VM, NM, and NV root tips, the data were cross-tabulated into a contingency table and the chi-square test of independence was performed. The cells in the contingency table, which were responsible for the significant departure from the independence of the examined variables were identified as those for which the absolute maximum of Pearson’s residual exceeded the value of 2.

The species diversity for each class of trees was estimated with the Chao1 and Shannon diversity indices. The differences in the soil samples’ characteristics between the tree classes were examined with the one-way analysis of variance (ANOVA) or the non-parametric Kruskal-Wallis test. The Kruskal-Wallis analysis was applied in the case of the soil parameters which that did not fulfil the the ANOVA’s assumptions: the homogeneity of variance (Levene’s test) and/or normality (Shapiro-Wilk test). In the case of significant differences, Tukey’s honestly significant difference (HSD) test (for ANOVA) and Dunn’s test (for Kruskal-Wallis) were used to identify the homogeneous groups of tree classes. Spearman’s correlation and principal component analysis (PCA) were used to relate the soil characteristics with the abundance of VM, NM, and NV root tips. The Kaiser-Meyer-Olkin (KMO) measure of sampling adequacy was applied to select the variables applicable for the PCA with the KMO threshold value equal to 0.6. Bartlett’s sphericity test was then used to confirm that the set of selected variables is suitable for structure detection.

The differences in the mycorrhizal community composition between the four damage classes of trees were illustrated using the non-metric multidimensional scaling (NMDS) ordination technique. The significant differences in the community composition across the damage gradient were tested with the PERMANOV after positive testing the multivariate homogeneity of group variances. The species data were transformed using the Hellinger transformation prior to the above analysis and the Bray-Curtis dissimilarity was applied.

Finally, the abundance bar plots of the observed ECM fungi species in different damage classes and in the individual trees were created.

## Results

**Fig 1:**
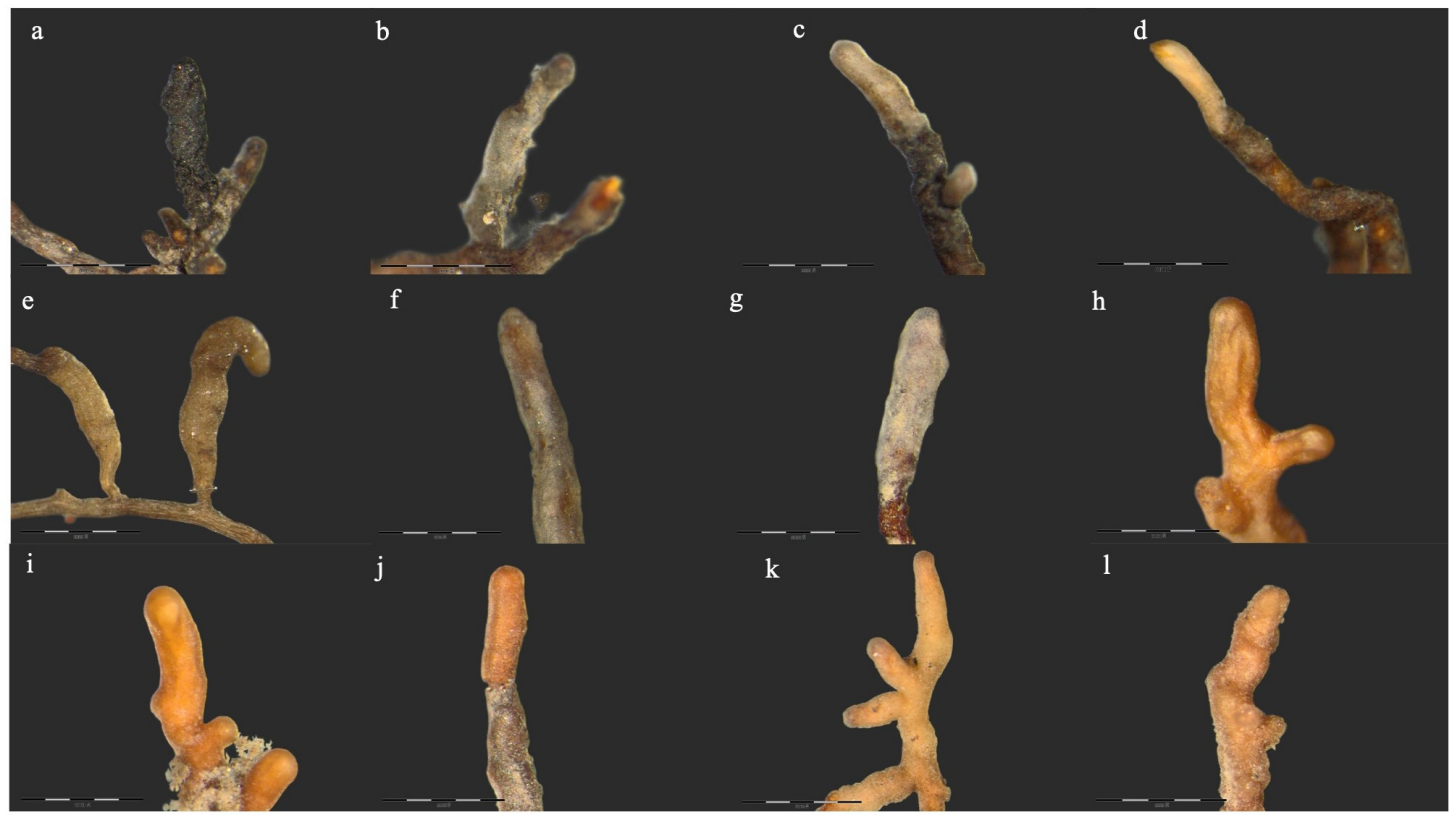
Ectomycorrhizas observed on linden trees in Gdańsk. (a) *Cenococcum geophilum* Fr., (b) *Hebeloma sacchariolens* Quél., (c) *Inocybe cincinnata* (Fr.) Quél., (d) *Inocybe grammopodia* Malençon, (e) *Inocybe maculata* Boud., (f) *Inocybe* manukanea (E. Horak) Garrido, (g) *Inocybe pelargonium* Kühner, (h) *Sebacina cystidiata* Oberw., Garnica & K. Riess., (i) *Tuber borchii* Vittad., (j) *Tuber rufum* Pollini, (k, l) *Tylospora asterophora* (Bonord.) Donk. Bars in each photograph indicate 0.4 mm length.

**Fig 2:**
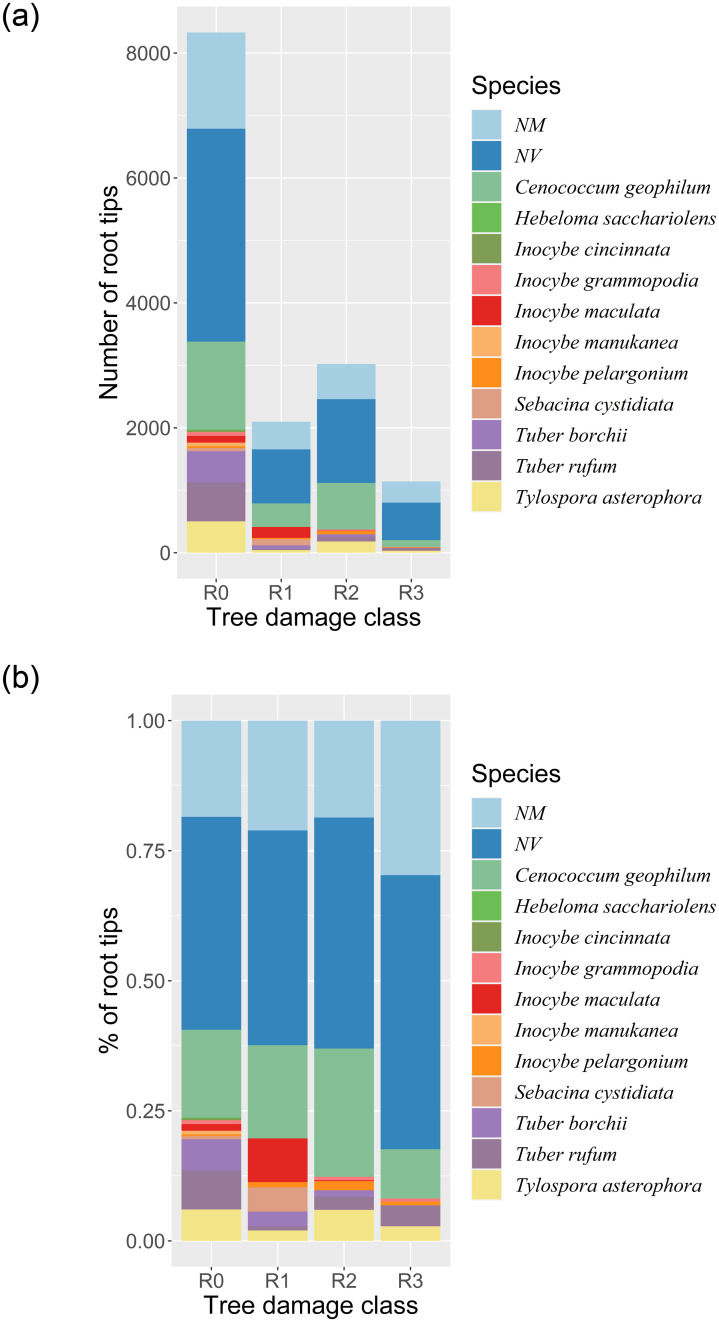
(a) The abundance of ectomycorrhizal, non-vital (NV) and non-mycorrhizal (NM) root tips for trees of different damage classes. Each colour represents the number of the root tips with the observed fungi species. (b) The percentage of ectomycorrhizal, non-vital (NV) and non-mycorrhizal (NM) root tips for trees of different damage classes. Each colour represents the percentage of the root tips with the observed fungi species.

The smallest degree of mycorrhization was observed in class R3 (18%). From mycorrhizal root tips after regrouping and combining based on the results of the molecular analysis, finally 11 fungal taxa were finally detected and assigned to a species level (Table 1, Fig 1). The five ECM fungal species (*Tylospora asterophora* (Bonord.) Donk, *Inocybe grammopodia* Malençon, *Inocybe pelargonium* Kühner, *Cenococcum geophilum* Fr., *Tuber rufum* Picco) were shared among all four trees damage classes (Table 1). *Cenococcum geophilum* was found to be the most abundant and frequent ECM fungal species among all classes (Fig 2a). Moreover, *C*. *geophilum* was present in more than 60% of all ECM tips (Fig 2b). For each damage class, the species composition of ECM fungi, fungal species richness and diversity indexes were analysed. The number of observed root tips decreased, from the R0 class trees, through the successive tree groups of increasing damage level. Taxa richness decreased similarly. The numbers of observed ECM root tips in trees from the R0, R1, R2 and R3 groups were 5956, 4472, 3022 and 1163, and the numbers of species were 10, 8, 7 and 5, respectively. For the individual trees, the numbers of observed root tips and the numbers of species were highly correlated: Spearman’s correlation was equal to 0.77 at p-value<0.0001. Due to lack of singletons and doubletons observed in the analysed samples, the Chao1 index computed for each tree class equalled the taxa richness.

**Table 1:** Estimated species richness, diversity and occurrence of fungal taxa associated with the roots of linden trees.

	BLAST top-hit			Tree vitality according to Roloff’s classification							
Identification	Closest match	NCBI	Identity [%]	R0		R1		R2		R3	
				Freq.	Abun.	Freq.	Abun.	Freq.	Abun.	Freq.	Abun.
**Basidiomycota**											
*Tylospora asterophora*	*Tylospora asterophora*	-	97	60	4.8	91	5.8	20	6.0	56	2.8
*Inocybe maculata*	*Inocybe maculata*	MT431581	99	50	1.3	36	4.6	10	0.2	-	-
*Inocybe grammopodia*	*Inocybe grammopodia*	MT431580	97	40	0.6	27	0.7	10	0.7	22	0.6
*Inocybe pelargonium*	*Inocybe pelargonium*	MT431583	97	20	0.3	36	0.7	30	1.8	11	0.7
*Inocybe cincinnata*	*Inocybe cincinnata*	-	97	30	0.5	-	-	-	-	-	-
*Inocybe manukanea*	*Inocybe manukanea*	MT431582	97	20	1.0	-	-	-	-	-	-
*Hebeloma sacchariolens*	*Hebeloma sacchariolens*	MT431579	97	10	0.3	-	-	-	-	-	-
*Sebacina cystidiata*	*Sebacina cystidiata*	MT431584	97	-	-	18	3.3	-	-	-	-
**Ascomycota**											
*Cenococcum geophilum*	*Cenococcum geophilum*	MT431587	99	90	17.7	82	16.2	90	24.7	67	9.3
*Tuber rufum*	*Tuber rufum*	MT431586	97	70	9.4	36	1.9	60	2.5	33	4.0
*Tuber borchii*	*Tuber borchii*	MT431585	97	60	4.0	45	7.2	40	1.2	-	-
Mycorrhizal fungal species richness [n]				10		8		7		5	
Degree of mycorrhization [%]				39.7		40.4		40.0		17.3	
**Chi-square test of independence (p-value<0.0001)**			Mean								
NV			43%	41%		40%*		44%		52%**	
NM			20%	19%		19%		19%		30%**	
VM			37%	40%*		41%**		37%		18%**	
Sum			100%	100%		100%		100%		100%	
**Estimated species richness**											
Chao-1				10		8		7		5	
**Diversity**											
Shannon diversity index (Hʹ)—for combined samples				1.57		1.69		1.10		1.21	
Shannon diversity index (Hʹ), mean of individual samples				0.90 ± 0.50		0.76 ± 0.38		0.56 ± 0.36		0.39 ± 0.45	

Data are the frequency (Freq.; percentage of colonised plants) and abundance (Abun.; percentage of mycorrhizal roots colonised) of fungal taxa on root tips. The contingency table for the tree class vs the abundance of VM, NM, and NV root tips is presented, with a percentage of each tree class samples for a given type of root tips. The cells responsible for the significant departure from the independence of the examined variables are indicated in bold. The significant difference was found with ANOVA between the values of the Shannon index at the p-value = 0.063. Pearson residuals analysis:

*—residuals exceeding 2,

**—residuals exceeding 3.

**Fig 3:**
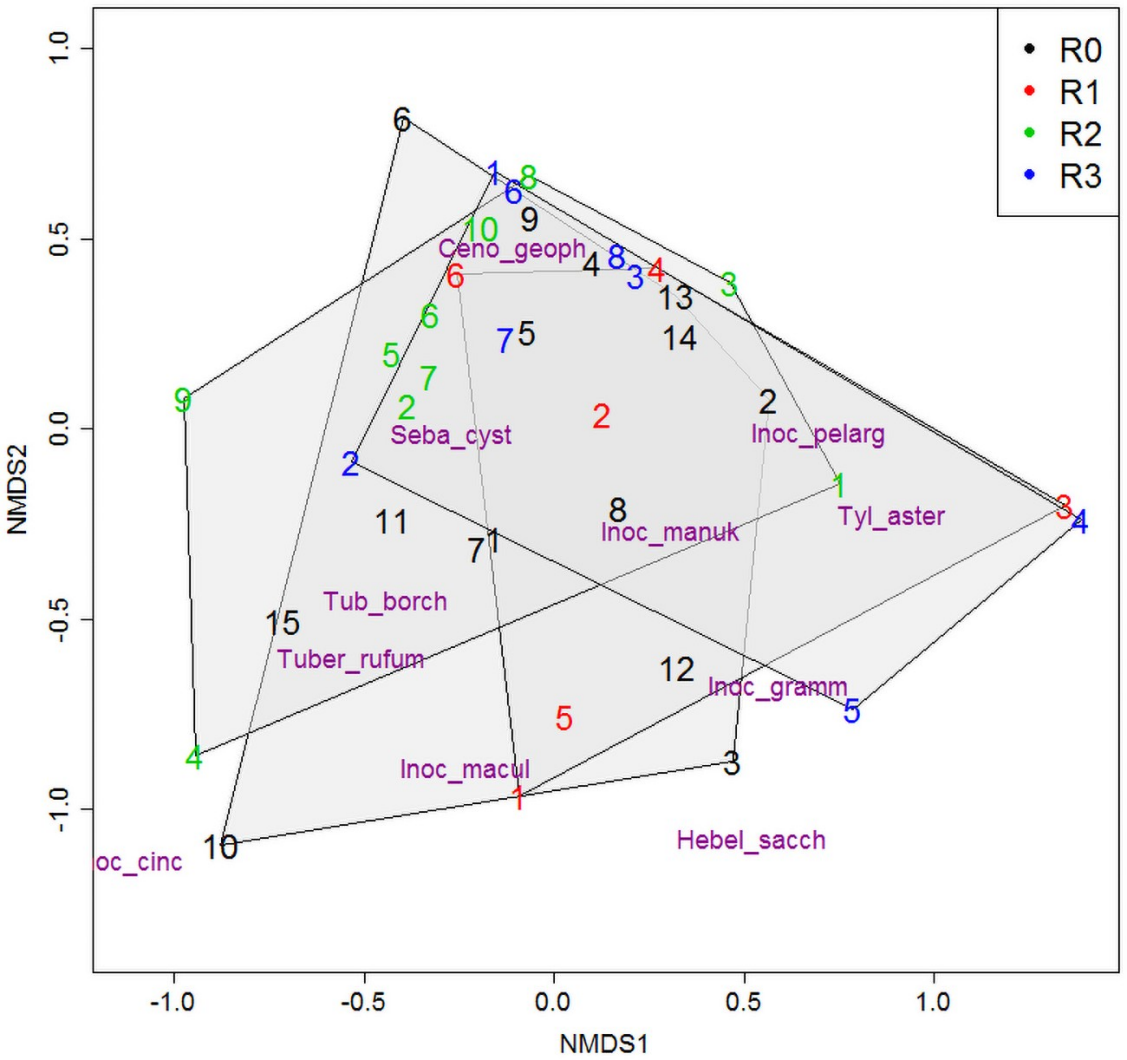
Non-metric MDS of the trees within Rolooff’s classes based on the ECM community. Convex hulls were drawn around each of the tree classes.

The NMDS ordination of the composition of the fungal communities in the examined trees is shown in Fig 3 (stress value 0.15). There is no indication in the chart that any species are assigned to any particular Roloff class. The plot confirms that the trees from classes R0 and R1 are richer in taxa, as their representatives lie mostly in the centre area of the chart, whereas the trees from classes R2 and R3, are more often located on the edges of the chart area, hence are linked with a smaller number of fungi species. These results are confirmed by the plots of the abundance of the observed ECM fungi species in individual trees from different damage classes (see S1a–S1d Fig). Finally, the PERMANOVA test showed no significant difference in the composition and relative abundances of fungi of different species in tree samples from different Roloff classes.

Nearly half of the tested tip samples, 43% were non-vital, while 20% and 37% of the samples belonged to the NM and VM types (Table 1). The chi-square test showed that, in comparison to this average distribution of the tip classes, the R0 class trees showed a slight excess of the VM type tips, trees from the R1 group showed an excess of the NV and VM type tips, and samples from the R3 class trees had strong overrepresentation of the NV and NM tips and underrepresentation of the VM class tips.

Mean values of the soil parameters between classes are compiled in Table 2. In the case of 6 soil characteristics, out of 16 examined, significant differences were found. These parameters were Cl, Na, Pb, Ca and Fe contents and the soil pH. Soil samples collected in the vicinity of trees in the R0 class had significantly lower pH and Na, Cl and Pb content than other soil samples. Considerable differences were observed between the content of Ca and Fe. In the first case, there was a significant difference only between the trees from the R0 and R3 damage classes, with the R0 class trees having the lowest Ca content. In the case of Fe, there was a significant difference between the trees from the R0, R1 and R3 damage classes, with the R0 class trees having the highest Fe amount. Also in this case, the average Fe content in the samples from the R2 tree class was much lower than in the case of the R0 class trees, but no significant differences were reported due to large variability of the R2 samples. Although the differences between means seem large, in some cases (Cr for example), though the differences between means seem large, no significant differences were found due to high variability of the data, especially among the R2 tree class samples.

**Table 2:** Mean values of selected physical and chemical properties of soil in samples associated with the trees of different damage classes.

	Tree vitality according to Roloff’s classification				Spearman correlation	
	R0	R1	R2	R3	# of root tips	VM
**pHH_2_O**	**6.9b**	**7.6a**	**7.8a**	**7.9a**	-0.55	-0.45
**Na (mg/l)**	**27.1b**	**132.3a**	**352.3a**	**290.0a**	-0.6	-0.49
**Cl (mg/l)**	**20.0b**	**34.5a**	**95.2a**	**56.4a**	-0.55	-0.43
**Pb (mg/kg)**	**55.9b**	**115.2a**	**145.6a**	**129.0a**	-0.35	-0.48
**Ca (mg/l)**	**1220.3b**	**1736.7ab**	**1523.6ab**	**1853.7a**		-0.34
**Fe (mg/l)**	**96.3a**	**63.0b**	**62.6ab**	**58.9b**		
N-NO_3_ (mg/l)	29.4	23.2	22.9	22.3	0.45	
C-org (%)	2.8	2.6	2.9	2.5		
Cr (mg/kg)	17.0	24.3	51.6	37.2		
Cu (mg/l)	7.0	9.3	11.5	13.7	-0.40	-0.45
Zn (mg/l)	27.1	37.2	43.5	32.1		-0.35
K (mg/l)	151.1	153.8	115.3	100.0	0.4	
Mg (mg/l)	122.7	130.7	99.4	104.4		
N-NH_4_ (mg/l)	11.3	11.0	10.0	6.9	0.32	
P (mg/l)	67.9	53.0	53.9	39.9		
Mn (mg/l)	2.0	2.2	2.6	2.7		

The soil features significantly different, at p-value<0.05, among the tree groups are in bold. Different letters indicate significant differences. Significant, at p-value<0.05.

Only some of the examined soil parameters were related to the abundance of the root tips and degree of mycorrhizal colonisation (Table 2). As can be seen, an increase of three soil parameters, N-NO_3_, N-NH_4_ and K, leads to an increase in the number of root tips. All the remaining soil features negatively influence the abundance of the root tips and the relative abundance of the mycorrhizal root tips (VM).

**Fig 4:**
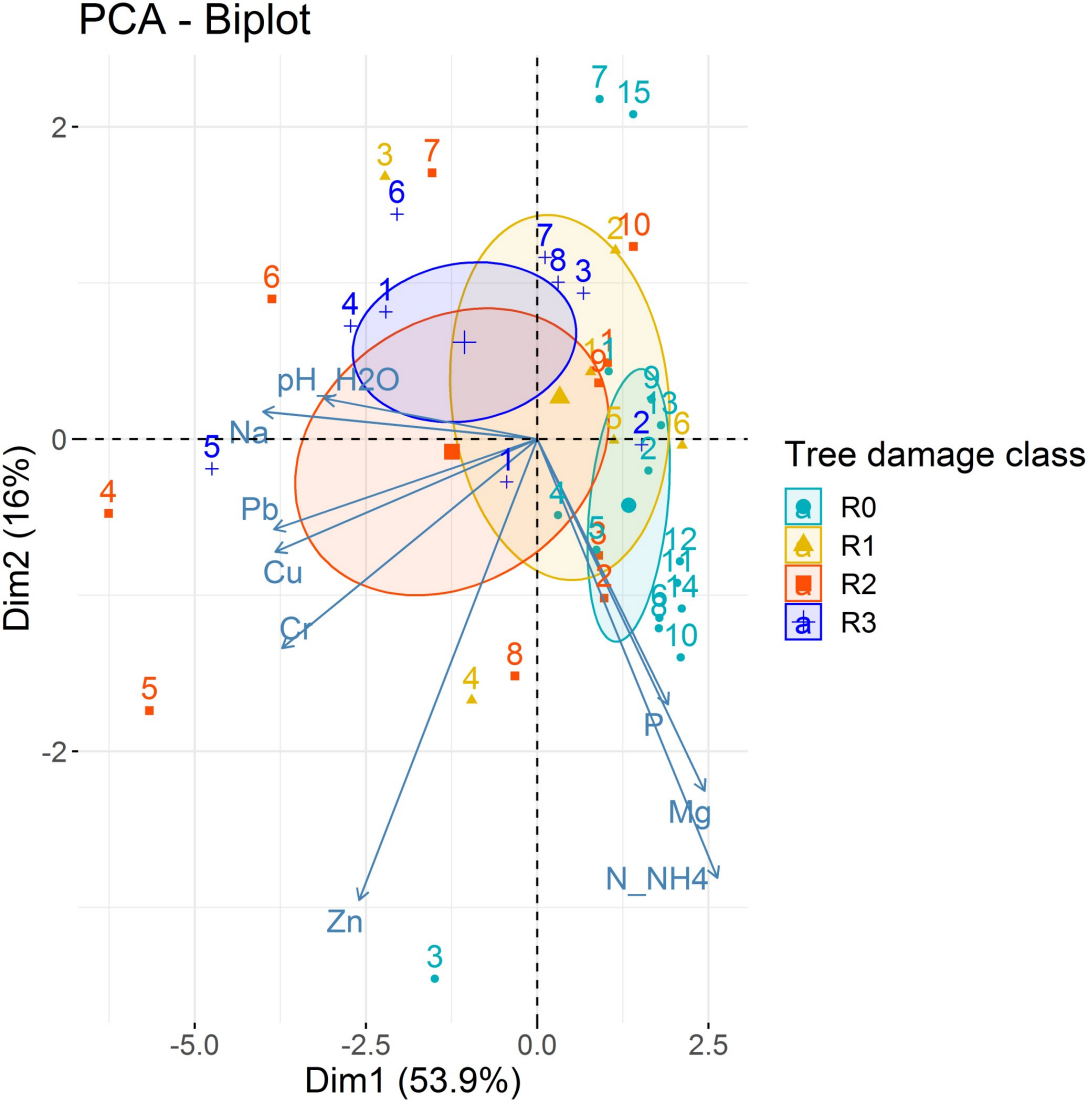
PCA plot representing the relationship between the soil parameters and trees of different Roloff’s classes. The ellipses are the 95% probability confidence ellipses around the mean point of each tree class.

The overall high variability of the soil characteristics in the samples related to the individual trees can be seen in the PCA plot in Fig 4. Correlation of the examined soil parameters allowed two main groups of them to be distinguished. The first group contains C-org, Cl, Cr, Cu, Na, Pb and Zn, and the second contains K, Mg and N-NH_4_. All second group members were negatively related to some representatives of the first one, namely Cu, Cr, Na and Pb. The parameter which links the two groups was pHH_2_O, positively correlated with Cu, Cr, Na and Pb and negatively with N-NH_4_. In the case of the R0 class trees, the samples are distributed parallel to the second group of soil parameters—Mg, K and N-NH_4_—and in the case of the other class trees, the high variability. The remaining soil features, Ca, Fe, P and N-NO_3_, had a weaker correlation with other parameters and Mn showed no relation to any parameter.

## Discussion

The study presented here investigated the relationships among the urban linden trees’ health status using Roloff’s classification with ECM communities. So far, few studies have dealt with the ECM community in urban linden trees [22, 36–38]. Considering the mycorrhization degree, only class R3 had significantly fewer vital ectomycorrhizal tips than other classes. These data confirmed the data obtained by studying the English oak trees [39], where fine roots of most declining trees had a lower proportion of vital and ectomycorrhizal tips. Moreover, among-class comparison suggested a relationship between tree health and abundance of all roots, with the highest proportion of non-vital root tips recorded in the most damaged stand (Table 1).

The *Tilia* species analysed in our study belong to Great Linden Avenue, which is subject to legal protection. Our study showed that the ECM community structure is highly dependent on the level of the linden tree decline. The observed ECM fungal species diversity differed significantly across the tree vitality. A similar study conducted in Italy, comparing the health situation of linden trees, classified as ‘moderately declining’ and ‘strongly declining’, showed that the number of ECM fungal species was lower in this second group in comparison to the first [36]. Since lack of nutrients, attack from pathogens, drought and use of de-icing salts are among the main causes of damage of urban trees [40], ECM fungi of urban trees may enhance their growth and survival in the urban environment. In our study the trees were already 60 years old and the mycorrhizal fungal population associated with their roots was likely to be well adjusted to the urban habitat. *Tilia* roots in R0 tree class harboured a diversity of ectomycorrhizal fungi. The number of 10 mycorrhizal morphotypes found in the R0 trees in this study was similar to the 12–13 morphotypes observed by Nielsen and Rasmussen [41] in native and planted forests in Denmark. The higher diversity of ectomycorrhizal fungi in the R0 group may be the result from the lower soil pH in comparison to other classes, and also partly due to the higher diversity of other ectomycorrhizal plants surrounding the *Tilia* trees in ‘park’ habitat compared to the ‘street’ habitat.

As hypothesised, a gradual increase in taxa richness was observed from the highest damage of trees (R3: 5 taxa) to the best health condition of trees (R0: 10 taxa). The increasing richness of ECM communities with increases in tree health may have been influenced by increases in the abundance of fine root tips (see S2 Fig). There are two mechanisms that could cause this: (i) sampling effort effect (more root tips = detection of more ECM fungi; (ii) niche availability effect (more root tips = more resources for ECM fungi = more diversity). The differences among the ECM fungal communities harboured by linden trees on the studied sites may be affected by salinity and concentration of heavy metals. The salt applied to roads in winter is a serious cause of urban tree damage [42], including water deficit, soil compaction, ion toxicity and ion imbalance [43, 44]. Moreover, Na and Cl may inhibit enzymatic activity of fungi [45]. In our study, the elevated amount of Na and Cl was the soil feature unique to R1, R2, and R3 tree classes when compared with the R0 tree class. The soil microbial communities are affected more by salinity than by extremes of any other abiotic factor [46], so this factor could have affected the lower species composition of the ECM fungi associated with the linden in R1, R2 and R3 classes.

The PCA analysis showed a gradual shift in the similarity between the adjacent damage classes (Fig 4). In part, these differences were due to a significantly lower concentration of heavy metals (e.g. Pb) in soil samples collected in the vicinity of R0 trees compared to other tree classes. In general, increased concentrations of heavy metals in the soil are known to negatively affect biodiversity [e.g. 47, 48]. Heavy metals cause damage to proteins, lipids and DNA [49]. Turpeinen, Kairesalo and Haggblom [50], who investigated the impact of heavy metal contamination on microbial communities, found a negative effect of metal pollution on fungal diversity. This is consistent with the findings in our study, where the R0 tree class was shown to host a higher ECM fungal richness than the other tree classes. On the other hand, Van Geel et al. [37] reported that the variability in ECM communities of *T*. *tomentosa* urban trees was little attributed by heavy metal pollution. It is important to note that Van Geel et al. [37] used high-throughput sequencing (HTS) as the basis of taxa identification and the results featured only mycorrhizae identified at the family level. A different sampling area was another point in the comparison of the results in the studies. The study of Van Geel et al. [37] was performed on a relatively large scale, due to its location in three European cities. In our study, we concentrated on one city and one street, which limited the potential for replication. More research is needed on a larger sample to reliably identify the reasons for the differences observed between our results and previous research.

Surprisingly, we also found several ECM common to all damage classes. There were genera belonging to early-stage fungi, including *I*. *grammopodia* and *I*. *pelargonium*. These fungi are often found in habitats with limited nutrient availability [51], for instance in urban ecosystems. Although *T*. *rufum* needs a more stable habitat [52], this fungus was abundantly present in all damage classes. It may have resulted from the alkaline conditions in soil, because Tuberaceae generally prefer more alkaline conditions [53]. This result may also suggest that either some genotypes are adapted to urban conditions or they are not outcompeted.

The ECM fungal species that we found to be predominant–*C*. *geophilum*–was present among all damage classes. The dominance of *C*. *geophilum* was not a surprising result, because this fungus is known as the most efficient drought-tolerant type [12, 54]. Considering the ECM community composition related to plant health status, Timonen and Kauppinen [22] demonstrated that *Cenococcum* spp. were more dominant in the roots of unhealthy street trees. The fungus *Cenococcum geophilum* is well known as a ubiquitous ectomycorrhizal symbiont mainly due to its pioneering capabilities and persistence of sclerotia in the soil [55]. Ectomycorrhizae of *C*. *geophilum* indicated active growth at low soil temperature and drought tolerance [12]. The abundant colonisation of roots by this species, in our study, might be to the low water resources. However, this interpretation, however, is made cautiously because the ECM community of urban trees in water stress were not studied. *Hebeloma sachariolens* was found only in soil samples from the R0 tree class where the content of N and P was higher than in other soil samples, which is in agreement with the findings of many authors [56–59] regarding the ability of this fungus species to tolerate rather high nutrient conditions. The formation of mycorrhizae by *T*. *borchii* and *T*. *maculatum* is hardly surprising as the fungi have been reported to form ectomycorrhizal symbioses with *Tilia* spp. elsewhere in Europe [22, 60, 61].

Overall, our results showed that the tree vitality was significantly associated with soil characteristics, especially with heavy metal pollution. Our knowledge of ECM communities in urban areas is still limited, and these findings provide new insights into ECM distribution patterns in urban ecosystems. Given the multifunctional role of ECM in urban ecosystems, further research should also include manipulation of mycorrhizal communities in the field.

## Supporting information

S1 Fig a The percentage of ectomycorrhizal, non-vital (NV) and non-mycorrhizal (NM) root tips in individual trees from R0 damage class. Each colour represents the percentage of the root tips with the observed fungi species. b. The percentage of ectomycorrhizal, non-vital (NV) and non-mycorrhizal (NM) root tips in individual trees from R1 damage class. Each colour represents the percentage of the root tips with the observed fungi species. c. The percentage of ectomycorrhizal, non-vital (NV) and non-mycorrhizal (NM) root tips in individual trees from R2 damage class. Each colour represents the percentage of the root tips with the observed fungi species. d. The percentage of ectomycorrhizal, non-vital (NV) and non-mycorrhizal (NM) root tips in individual trees from R3 damage class. Each colour represents the percentage of the root tips with the observed fungi species.
(TIF)

S2 Fig Boxplot of distribution of ‘vital ECM’ (VM) ‘non-vital’ (NV) and ‘vital non-ECM’ (NM) root tips in groups of trees from the examined damage classes R0, R1, R2 and R3. Each box is drawn from the first to third quartile with the median denoted in the middle. The whiskers spread from the minimal to maximal value and the dots represent the outliers.
(TIF)

S1 Dataset (XLSX)
