# Supplementary figures and images for: The ectomycorrhizal community of urban linden trees in Gdańsk, Poland
Source: PLoS One. 2021 Apr 26;16(4):e0237551. doi: 10.1371/journal.pone.0237551 (PMC8075230; doi:10.1371/journal.pone.0237551)

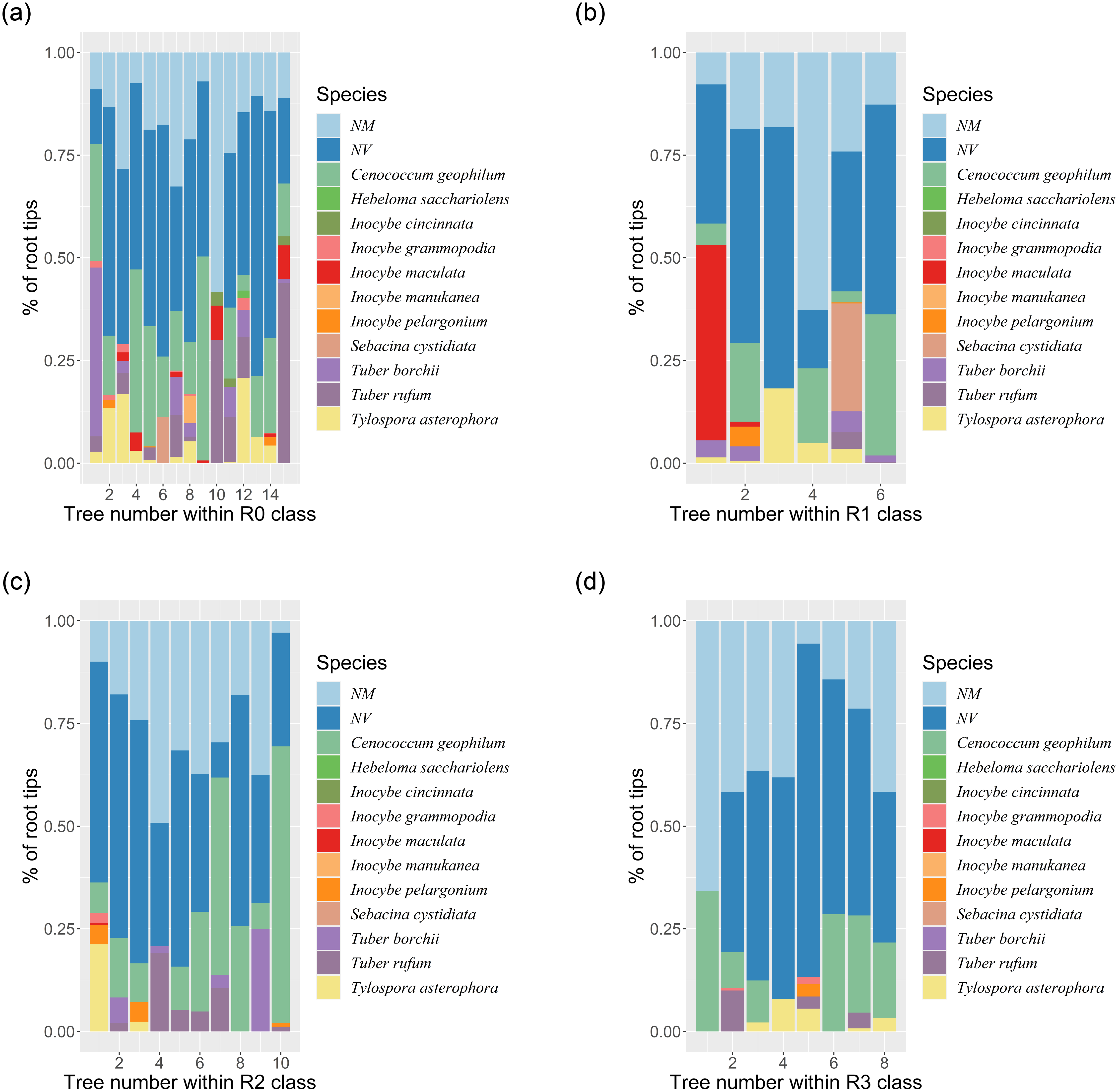

Supplement: S1 Fig — a The percentage of ectomycorrhizal, non-vital (NV) and non-mycorrhizal (NM) root tips in individual trees from R0 damage class. Each colour represents the percentage of the root tips with the observed fungi species. b. The percentage of ectomycorrhizal, non-vital (NV) and non-mycorrhizal (NM) root tips in individual trees from R1 damage class. Each colour represents the percentage of the root tips with the observed fungi species. c. The percentage of ectomycorrhizal, non-vital (NV) and non-mycorrhizal (NM) root tips in individual trees from R2 damage class. Each colour represents the percentage of the root tips with the observed fungi species. d. The percentage of ectomycorrhizal, non-vital (NV) and non-mycorrhizal (NM) root tips in individual trees from R3 damage class. Each colour represents the percentage of the root tips with the observed fungi species. (TIF) [file pone.0237551.s001.tif]

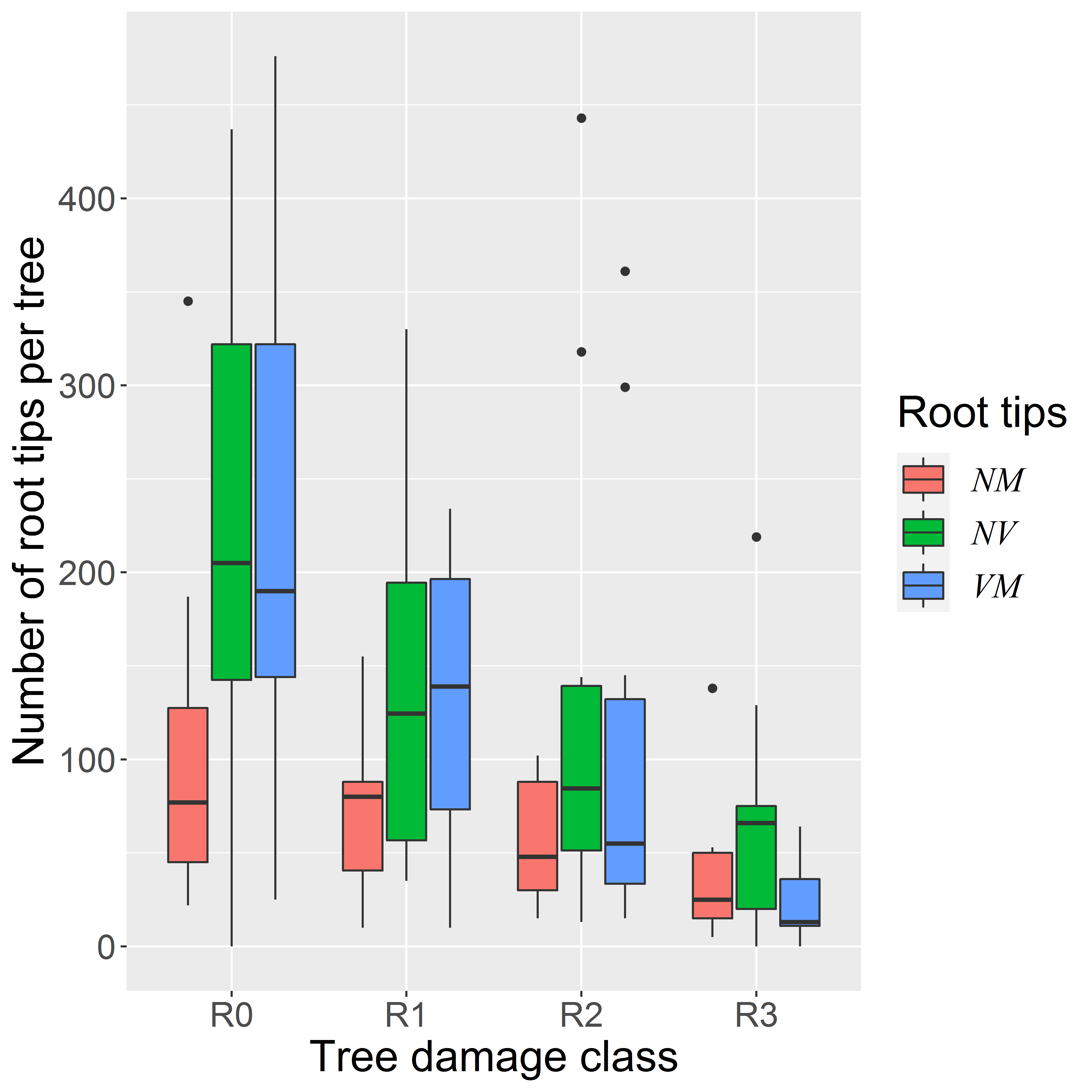

Supplement: S2 Fig — Each box is drawn from the first to third quartile with the median denoted in the middle. The whiskers spread from the minimal to maximal value and the dots represent the outliers. (TIF) [file pone.0237551.s002.tif]
